# Loss of SELENOF Induces the Transformed Phenotype in Human Immortalized Prostate Epithelial Cells

**DOI:** 10.3390/ijms222112040

**Published:** 2021-11-07

**Authors:** Lenny K. Hong, Shrinidhi Kadkol, Maria Sverdlov, Irida Kastrati, Mostafa Elhodaky, Ryan Deaton, Karen S. Sfanos, Heidi Wang, Li Liu, Alan M. Diamond

**Affiliations:** 1 Department of Pathology, College of Medicine, University of Illinois at Chicago, Chicago, IL 60612, USA; lennyh@uic.edu (L.K.H.); skadko2@uic.edu (S.K.); mariasve@uic.edu (M.S.); mehod@uic.edu (M.E.); rdeaton@uic.edu (R.D.); 2 Research Histology Core, Research Resources Center, University of Illinois at Chicago, Chicago, IL 60612, USA; 3 Department of Cancer Biology, Loyola University of Chicago, Cardinal Bernardin Cancer Center, Maywood, IL 60153, USA; ikastrati@luc.edu; 4 Department of Pathology, Johns Hopkins University School of Medicine, Baltimore, MD 21287, USA; ksandel1@jhmi.edu; 5 Department of Epidemiology and Biostatistics, School of Public Health, University of Illinois at Chicago, Chicago, IL 60612, USA; xwang298@uic.edu (H.W.); liliu@uic.edu (L.L.)

**Keywords:** prostate, cancer, selenium, selenoprotein, tumor suppressor

## Abstract

SELENOF is a member of the class of selenoproteins in which the amino acid selenocysteine is co-translationally inserted into the elongating peptide in response to an in-frame UGA codon located in the 3′-untranslated (3′-UTR) region of the SELENOF mRNA. Polymorphisms in the 3′-UTR are associated with an increased risk of dying from prostate cancer and these variations are functional and 10 times more frequent in the genomes of African American men. SELENOF is dramatically reduced in prostate cancer compared to benign adjacent regions. Using a prostate cancer tissue microarray, it was previously established that the reduction of SELENOF in the cancers from African American men was significantly greater than in cancers from Caucasian men. When SELENOF levels in human prostate immortalized epithelial cells were reduced with an shRNA construct, those cells acquired the ability to grow in soft agar, increased the ability to migrate in a scratch assay and acquired features of energy metabolism associated with prostate cancer. These results support a role of SELENOF loss in prostate cancer progression and further indicate that SELENOF loss and genotype may contribute to the disparity in prostate cancer mortality experienced by African American men.

## 1. Introduction

Prostate cancer remains a significant clinical problem in the US, with an estimated 248,530 men diagnosed with the disease and 34,130 men dying from prostate cancer in 2021, making death from prostate cancer the second leading cause of death among American men [1]. Moreover, genetic factors contribute to prostate cancer risk; individuals with first-degree relatives diagnosed with prostate cancer have a higher risk of disease (as confirmed by meta-analysis [2]). However, most men with prostate cancer do not have a known family history, which strongly suggests that high-risk genetic factors are likely to be of relatively low penetrance and may be influenced by environmental variables.

Epidemiological studies report an inverse association between selenium (Se) levels and risk of several cancer types [3]. There are 25 human selenium-containing proteins or selenoproteins containing selenocysteine [4]. One selenoprotein implicated in prostate cancer etiology is SELENOF [5] which was originally identified as a human T cell 15 kDa protein that labels with ^75^Se and is expressed at high levels in the prostate [6,7]. SELENOF belongs to the family of selenium-containing proteins in which selenium is inserted co-translationally in response to a UGA codon in the corresponding mRNA [8]. Notably, the function of SELENOF remains unknown. In most cell types, SELENOF resides in the endoplasmic reticulum (ER) in a complex with UDP-glucose: glycoprotein glucosyltransferase (UGGT), which helps to maintain correct protein folding [9]. However, SELENOF is uniquely associated with the plasma membrane in normal prostate epithelial cells [10].

The *SELENOF* gene is polymorphic in the 3′-untranslated region (UTR) that recognizes in-frame UGA codons as the amino acid selenocysteine [6]. Polymorphisms at positions 811 and 1125 form a haplotype where a C at 811 always corresponds to a G at 1125 and a T at 811 always corresponds to an A at 1125. Using two different specialized reporter constructs, these genetic variations were shown to be functional and likely determine the amount of SELENOF made as a function of selenium availability [7,11]. A potential role for SELENOF in carcinogenesis in multiple organ types is indicated by several lines of evidence, including loss of heterozygosity in breast cancers [11,12] and the association of specific polymorphisms of *SELENOF* and the risk of colorectal cancer [13,14]. Genetic data implicate SELENOF in prostate cancer etiology, with a statistically significant association between *SELENOF* polymorphisms, plasma selenium levels, and prostate cancer mortality [15]. Indeed, 811/1125 polymorphisms exhibit a trend toward association with prostate cancer-specific mortality.

Epidemiological data reported by Penney et al. [15] and our *in vitro* data [7,11] indicate an interaction between selenium and SELENOF, and its association with prostate cancer mortality. Selenium is distributed to the body from the liver as a component of selenoprotein P (SELENOP), previously referred to as SEPP1 [5], which contains 10 or more UGA-encoded selenocysteine residues. There are two functional polymorphisms in the gene for SELENOP, one a coding single-nucleotide polymorphism (SNP) (rs3877899, ^Ala^234^Thr^) that influences plasma selenium levels [16] and another in the 3′ UTR that effects SELENOP synthesis (rs7579). These polymorphisms interact with *SELENOF* alleles to impact prostate cancer risk in a cohort of European men [17].

Studies using human tissues have supported a role for the loss of SELENOF in prostate cancer etiology. SELENOF localizes to the plasma membrane in normal human prostate tissues, but not in other tissues, where its location is predominately in the ER and levels are dramatically reduced in prostate cancers compared to adjacent benign tissue [10]. The Gleason Grade Group, an indicator of prostate cancer aggressiveness, negatively associates with *SELENOF* genotype [10]. While these data provide an indication of a contributing role for SELENOF loss in prostate cancer incidence or severity, the studies presented here were designed to obtain evidence that the loss of SELENOF may directly contribute to disease progression.

## 2. Results

### 2.1. Lower Levels of SELENOF in Human Prostate Tissues Obtained from African American and Caucasian Men

**Figure 1:**
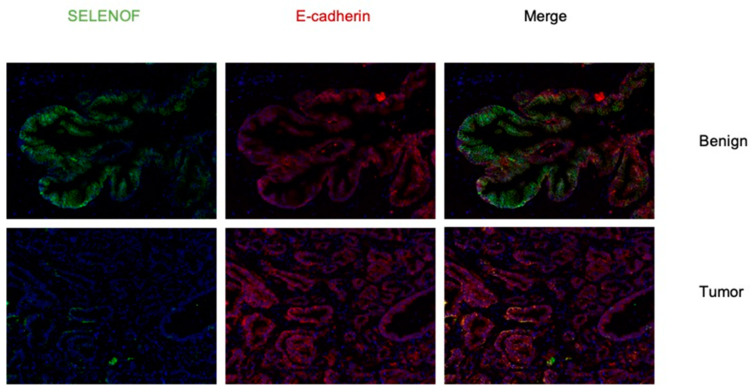
SELENOF levels are lower in prostate cancer compared to benign prostatic tissue. A representative immunofluorescent image of SELENOF (green) and E-cadherin (red). The top images show a region of benign prostatic tissue and the bottom images represent a region of prostate cancer. (Magnification = 20×). Staining with anti-E-cadherin antibodies was included both as a marker for the outer membrane and to restrict quantification of images to epithelial cells.

Previously, we reported that lower levels of SELENOF were found in prostate cancer of African Americans compared to that from Caucasian men [10]. Here, a disparity TMA set was obtained from the Prostate Cancer Biorepository Network (PCBN) containing primarily high-grade tumor and matched benign prostate tissue cores (4 tumor and 4 benign tissue cores) from 60 Caucasian and 60 African American men matched on age +/− 3 years, Gleason grade, and stage. The TMA slides were stained using a validated SELENOF specific antibody to determine SELENOF levels, an E-cadherin antibody to identify regions of epithelial cells and to designate membrane localization, and DAPI to stain the nucleus of each cell. Only areas of the obtained images with both E-cadherin and SELENOF were used in the analysis to exclude stromal regions and non-specific background signal. As we published previously [10], SELENOF was expressed mainly in the epithelial cells and not in the stromal cells and was located near the lateral and basal membrane of the epithelial cells with undetectable levels on the apical side facing the lumen. Prostate cancer had dramatically lower levels of SELENOF compared to adjacent benign tissue with diffuse staining throughout the cytoplasm (See Figure 1 for representative images).

The comparisons of SELENOF intensities between benign and tumor-appearing regions, and between races are listed in Supplemental Table S1. We analyzed averaged SELENOF levels, and the relative amount or percentages of membrane associated SELENOF, calculated as the amount of SELENOF that co-localized with E-cadherin, compared to that amount determined for the entire cell. Paired *t*-tests revealed that SELENOF levels and distribution were dramatically lower in cancer cells when compared to benign tissues, consistent with our previous study [10]. Differences in SELENOF levels that co-localized with E-cadherin, presumably at the outer cellular membrane, were analyzed by paired *t*-test for significance. SELENOF levels that did not co-localize with E-cadherin were considered cytoplasmic. Regardless as to whether signals obtained were from the membrane or cytoplasmic regions, epithelial cells from prostate cancer cores had significantly lower levels and percentages of SELENOF in the membrane than benign prostate cores (Supplemental Table S1, top).

To investigate if there was a difference in SELENOF levels between African Americans and Caucasians, race comparisons on log transformed SELENOF levels, the relative cellular distribution were performed for each tissue and for the benign versus cancer differences (Supplemental Table S1, bottom). There were no significant differences in SELENOF levels or membrane percentages in benign prostate tissues between Caucasians and African Americans, regardless of whether the entire cell, cytoplasmic, or membrane regions were considered. Contrary to the previous study indicating that prostate tissue from African Americans had lower levels of SELENOF compared to that of Caucasians [10], tissue from African Americans had significantly higher levels of SELENOF in prostate cancer cores compared to Caucasians. Possible reasons for the differences in these results include the different designs of the TMAs, the much smaller sample size for the TMA presented here and much fewer low-grade cancers in this analysis compared to the previous one. The higher levels of SELENOF in African American cancer cells were statistically significant whether the entire cell (*p* = 0.036), cytoplasmic (*p* = 0.036), or membrane (*p* = 0.024) regions were compared. Similar race differences were also found in the cytoplasmic (*p* = 0.047), membrane (*p* = 0.039) regions, and the entire cell (*p* = 0.036). The difference in SELENOF levels and distribution between benign and cancer cells were slightly higher in African Americans than Caucasian samples, although these differences were not statistically significant.

To assess if membrane-located SELENOF levels were associated with prostate cancer stage, and if race modified the association, multivariate ordinal logistic regression models for Gleason score were employed. In these models, the effects of SELENOF that co-localized with the outer membrane-located protein E-cadherin in terms of percentage, race, and their interactions were tested. Gleason grading is a scoring system used by pathologists based on histology [18]. Each prostate core is assigned a score ranging from 1 to 5, with 1 representing benign, well-differentiated glands, 3 representing moderately differentiated glands, and 5 representing poorly differentiated glands [18]. Two scores are reported with the primary score being the most prevalent Gleason grade in the biopsied core and a secondary score representing the second most prevalent Gleason grade [19]. The primary and secondary scores are added together to calculate the Gleason score [19]. Out of 60 African American and 59 Caucasian patients, 20 (16.8%) patients had Gleason grade of 3 + 3, 26 (21.9%) had 3 + 4 or 3 + 5, 19 (16.0%) had 4 + 3, and 54 (45.4%) had 4 + 4, 4 + 5, or 5 + 4. In the multivariate ordinal logistic regression models, Gleason grades 4 or higher were treated as an ordered categorical outcome SELENOF distribution (percentages) and interactions with race were examined separately as the SELENOF percent distribution in cancer and benign cells (Supplemental Table S2a), and benign minus cancer percentage differences (Supplemental Table S2b).

The model selection process of SELENOF distributions in benign and cancer cells, and interactions between race and cancer cell membrane distribution indicated significant associations with Gleason grades. Higher cytoplasmic levels decreased the risk of higher Gleason grades (OR = 0.92, *p*-value = 0.0003). Among Caucasian patients, higher membrane levels strongly reduced the risk of higher Gleason grades (estimate = −0.34, *p*-value = 0.0006; OR of Caucasian membrane association is 0.71). Such protective effects of membrane localization were significantly reduced in African American patients (estimate of interaction = 0.26, *p*-value = 0.0132). The effect of SELENOF in the membrane of cancer cells in African American patients had an OR closer to 1 compared to that in Caucasians (OR = 0.92, *p*-value = 0.015).

The model selection process for the ordinal logistic regression model using benign-cancer SELENOF percent differences, race, and their interactions as predictors for Gleason grades revealed race-differential effects of the benign-cancer difference in SELENOF percentages in the entire cell and in the cytoplasm (Supplemental Table S2b). Benign-cancer cell percent differences did not influence Gleason grade in Caucasians (OR = 0.98, *p*-value = 0.80). However, an increase in benign-cancer cell SELENOF percent difference in the entire cell significantly increased the risk of having higher Gleason grade for African Americans (OR = 1.57, 95% CI = 1.2, 2.04). These results indicated that African Americans were almost 1.6 times more likely to have a higher Gleason score with each percent increase in the difference in SELENOF levels between benign and prostate cancer. Similarly, the benign-cancer cytoplasmic percent difference did not influence Gleason grades among Caucasians (OR = 0.999, *p*-value = 0.99). However, an increase in the benign-cancer cell SELENOF cytoplasmic percentage difference significantly reduced the risk of having a higher Gleason grade for African Americans (OR = 0.59, 95% CI = 0.43, 0.80). In other words, among African Americans, each percent increase in the benign-cancer cytoplasmic difference resulted in and odds of 1/0.59 = 1.70 times in having a lower Gleason grade.

### 2.2. Reducing SELENOF Levels Alters Phenotypes Associated with Transformation

**Figure 2:**
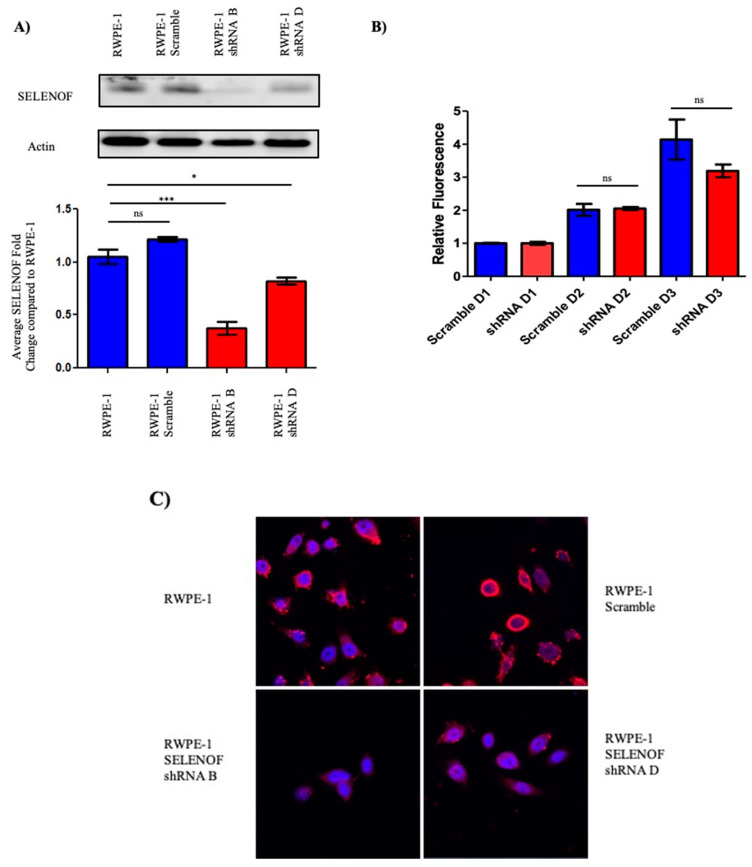
Reduction of SELENOF levels in RWPE-1 cells. (**A**) SELENOF levels were successfully reduced with an shRNA vector. Western blots using anti-SELENOF antibodies were quantified and a two-tailed *t*-test was performed for significant differences parental RWPE-1 cells and transfected RWPE-1 cells. *n* = 3, * *p* < 0.05, *** *p* < 0.001. (**B**) Fluorometric dsDNA quantification was performed after 3 days. Data are represented as the mean ± SEM, ns, not significant, *n* = 3. (**C**) Parental RWPE-1 and RWPE-1 scramble cells exhibit SELENOF membrane-associated localization shown in red, similar to what is seen in human benign tissues. Nuclei are stained blue with DAPI. Both shRNA RWPE-1 transfected clones have diffuse SELENOF staining in the cytoplasm similar to what is seen in prostate cancer tissue and prostate cancer cell lines (magnification is 63×).

To investigate whether the loss of SELENOF in prostate cancer indicates a tumor suppressor function, immortalized and non-transformed RWPE-1 human prostate cells were used because of their high expression of SELENOF and similar localization of SELENOF near the plasma membrane as seen in human benign tissues [10]. Four shRNA constructs and a scramble construct were obtained, transfected into RWPE-1 cells and stably transfected RWPE-1 cells were selected using puromycin. Pools and individual clones of SELENOF shRNA and SELENOF scramble transfected cells were isolated and expanded. As shown in Figure 2A, parental RWPE-1 cells and the pool of RWPE-1 cells transfected with the scramble shRNA construct exhibit high levels of SELENOF. Two of the SELENOF shRNA transfectant pools, designated shRNA B and shRNA D, displayed reduced levels of SELENOF by approximately 60% and 20%, respectively (Figure 2A). Fluorescent signals from the Western blots were quantified and presented as a fold change compared to the parental RWPE-1 cells below the corresponding Western blot. There was no significant difference in SELENOF levels between the parental RWPE-1 cells and the RWPE-1 scramble pool cells. SELENOF levels were significantly lower in shRNA B RWPE-1 cells and shRNA D RWPE-1 cells compared to the parental RWPE-1 cells (*p* < 0.001 and *p* < 0.05, respectively).

The reduction of SELENOF levels did not alter the proliferation of shRNA RWPE-1 cells relative to RWPE-1 scramble cells after 24, 48, and 72 h (Figure 2B). Given the localization of SELENOF in RWPE-1 cells near the plasma membrane [10], SELENOF was visualized by immunofluorescence to determine if any changes in localization occurred with the reduced SELENOF levels (Figure 2C). Both pools of RWPE-1 shRNA cells exhibited lower levels of SELENOF. SELENOF staining was in a diffuse pattern throughout the cytoplasm that was consistent with what was seen previously in prostate cancer tissues and prostate cancer cell lines [10]. With the greatest reduction in SELENOF achieved in the SELENOF shRNA B pool cells compared to either native RWPE-1 cells or an expanded clone transfected with a control, scrambled shRNA construct, further experiments utilized two individual clones derived from the shRNA B pool, each with a similar reduction in SELENOF as seen in the pool samples.

**Figure 3:**
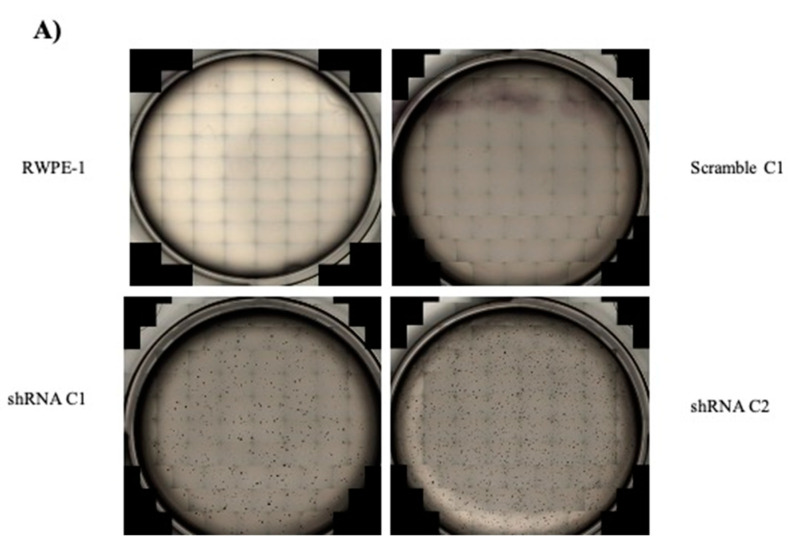

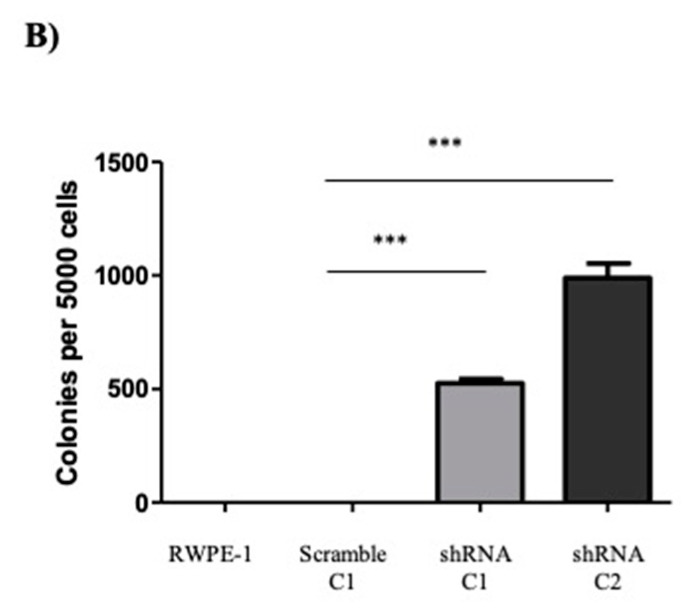
Reduction of SELENOF in RWPE-1 cells results in anchorage-independent growth. (**A**) Representative images of cells cultured in soft agar used to quantify the number of colonies formed. RWPE-1 and RWPE-1 scramble C1 did not form any colonies. (**B**) Quantification of the average number of colonies formed per 5000 cells plated. Data are represented in mean ± SEM, *n* = 3, and a two-tailed paired *t*-test was used to determine statistical differences *** *p* < 0.001.

To investigate if the reduction of SELENOF could alter the phenotype of parental RWPE-1 cells, RWPE-1 scramble, and two individual clones of shRNA RWPE-1 cells, were subjected to commonly used assays of cellular transformation. The ability of cells to grow in semi-solid media was assessed as anchorage-independent growth, a common phenotype of transformation [20,21,22]. Five thousand cells were seeded in semi-solid media, allowed to grow into colonies and imaged. As shown in Figure 3A, parental RWPE-1 and RWPE-1 scramble cells did not form any colonies in the semi-solid media. In contrast, clone 1 of the shRNA B RWPE-1 cells formed approximately 500 colonies per seeded 5000 cells and clone 2 of the shRNA B RWPE-1 cells formed approximately 1000 colonies per seeded 5000. These results indicated that reducing SELENOF levels in non-transformed RWPE-1 cells promoted anchorage-independent growth, a phenotype of transformation.

**Figure 4:**
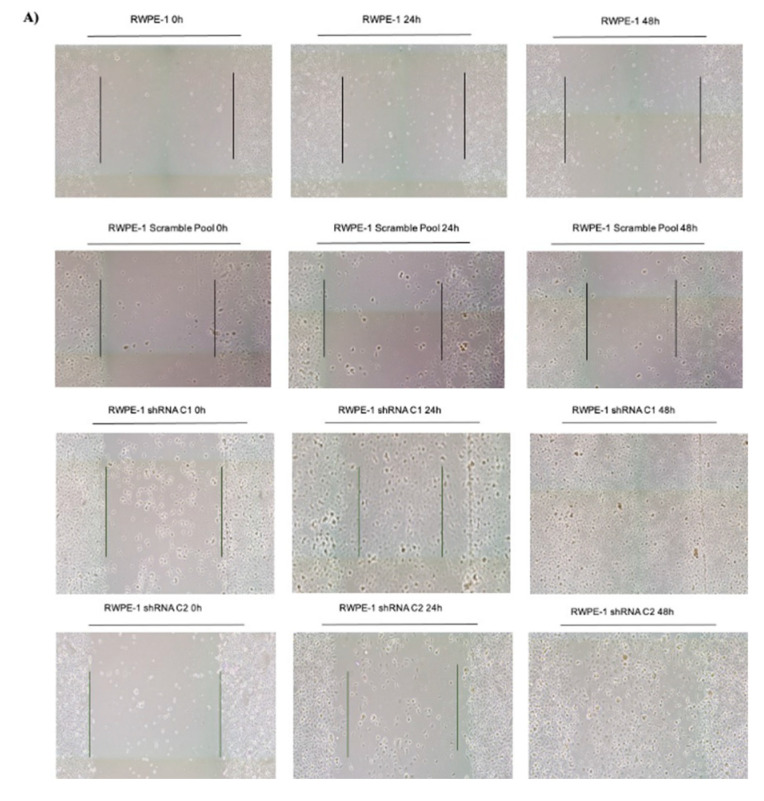

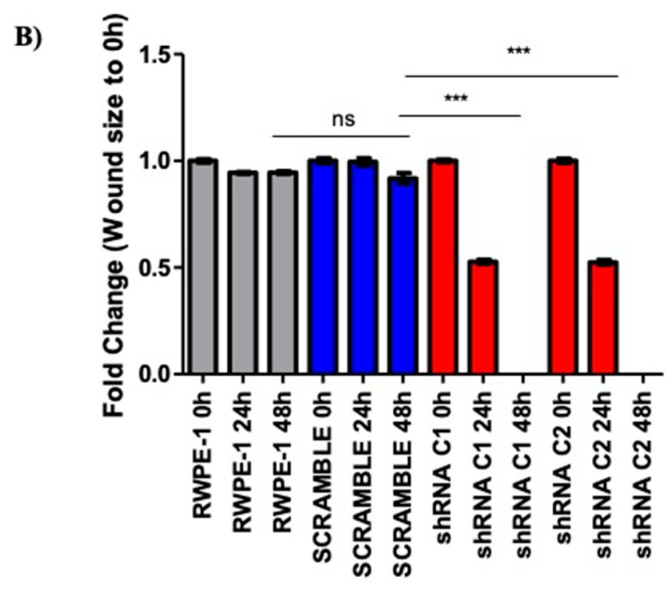
Reduction of SELENOF increases the migration of RWPE-1 cells in a scratch assay. (**A**) Representative images were captured at 0 h, 24 h, and 48 h for RWPE-1, RWPE-1 scramble, and 2 clones of RWPE-1 shRNA SELENOF cells. (**B**) Quantification of the scratch from three independent experiments are shown. The images were captured using the EVOS FL Auto Imaging system (ThermoFisher) and the width was measured using ImageJ. Data are represented as the mean ± SEM, ns, not significant, *n* = 3 and a two-tailed paired *t*-test was used to determine statistical significance, *** *p* < 0.001.

A scratch or wound healing assay was next used to determine the ability of cells to migrate on a tissue culture dish, a surrogate for aggressive or advanced cancer cells [23]. Parental RWPE-1, RWPE-1 scramble, and two clones of shRNA RWPE-1 cells were plated to form a fully confluent cell monolayer. A scratch was created using a pipette tip and the width of the scratch was measured. Although no differences in proliferation were observed when SELENOF levels were reduced, aphidicolin was used as an anti-proliferative agent to ensure differences in proliferation would not affect the results. Forty-eight hours after the initial scratch, both shRNA RWPE-1 clones nearly closed the wound completely while the parental RWPE-1 and RWPE-1 scramble-transfected cells only decreased the width of the wound slightly (Figure 4A). The widths of the scratch at 24 and 48 h were compared to the initial scratch width made at 0 h as a fold change (Figure 4). No significant difference was observed when the RWPE-1 scramble pool cells when compared to the parental RWPE-1 cells. Both shRNA RWPE-1 clones significantly migrated faster when compared to the RWPE-1 scramble cells at 48 h (*p* < 0.0001, Figure 4B).

### 2.3. Effects of SELENOF on Metabolism

**Figure 5:**
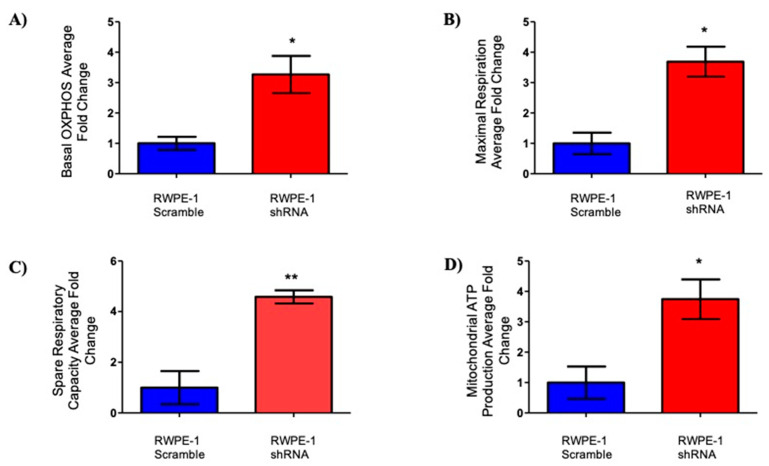
Reduction of SELENOF increases oxygen consumption in RWPE-1 cells. Average fold changes are represented for (**A**) basal, (**B**) maximal respiration after FCCP injection, (**C**) spare respiratory capacity (difference in peak OCR and basal measurements), and (**D**) mitochondrial ATP production (difference before and after oligomycin injection). Data are presented as the mean ± SEM, *n* =3, two-tailed paired *t*-test, * *p* < 0.05, ** *p* < 0.01, *n* = 3.

The metabolism of the benign prostate includes a truncated TCA cycle with glycolysis predominating to accumulate high levels of citrate required for sperm health [24]. Prostate carcinogenesis often involves a shift in energy metabolism from glycolysis to oxidative phosphorylation [25,26]. To investigate the effects of SELENOF on mitochondrial respiration in prostate cells, the oxygen consumption rate (OCR) of RWPE-1 cells with reduced SELENOF and control cells were measured in real time using the Seahorse XFe24 platform. The OCR is presented as the fold change of shRNA RWPE-1 cells compared RWPE-1 scramble cells in Figure 5. Reducing the levels of SELENOF significantly increased basal oxidative phosphorylation by approximately 3-fold (Figure 5A) compared to control RWPE-1 scramble cells. Maximal respiration is calculated by measuring the OCR after ATP synthase is inhibited by oligomycin and an uncoupler, FCCP, is injected into the assay. The spare respiratory capacity is the difference between the peak of OCR after FCCP injection and the initial basal OCR. Reducing the levels of SELENOF in RWPE-1 cells resulted in increased maximal respiration and spare respiratory capacity by 3.5-fold (Figure 5B) and 4-fold (Figure 5C), respectively. ATP production (Figure 5D), which is determined as the difference in OCR before and after oligomycin injection that inhibits ATP synthase also increased by 3-fold. Together, these results indicate that the reducing SELENOF levels in RWPE-1 cells increase mitochondrial respiration and presumably ATP synthesis.

**Figure 6:**
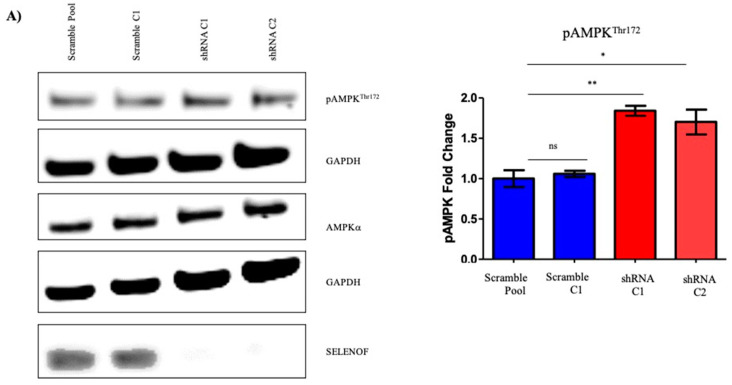

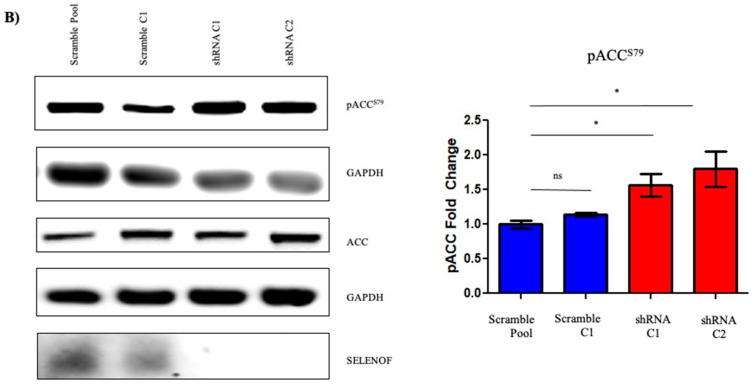
Reducing SELNENOF levels resulted in enhanced phosphorylation of AMPK and ACC in RWPE-1 cells. (**A**) Representative Western blots using anti-SELEONOF antibodies demonstrating the increase in the phosphorylation of AMPK in shRNA RWPE-1 cells without changes in total AMPK. (**B**) Representative Western blot and quantification of the changes in the phosphorylation of ACC and total levels of ACC. Florescent intensities were quantified and are shown next to the corresponding Western blots of three independent biological replicates. The data are presented as the mean ± SEM, *n* = 3, ns, not significant, * *p* < 0.05, ** *p* < 0.01.

A recent study using hepatic cells isolated from SELENOF knockout mice indicated differences in the levels of proteins related to energy metabolism, specifically in pathways involving glycolysis/gluconeogenesis and the TCA cycle compared to control mice [27]. In addition, SELENOF knockout mice experienced a greater frequency of glucose and lipid metabolism disorders [28]. Given these observations, changes in AMP-activated protein kinase (AMPK) in SELENOF knock down cells were explored due to its central role in cellular metabolism. AMPK is activated by phosphorylation at Thr172 when ATP levels decline resulting in the stimulation of glucose utilization for ATP production. Reducing SELENOF in RWPE-1 cells resulted in an approximately 1.7–2-fold increase in pAMPK levels without changes in total AMPK (Figure 6A). Both a representative Western blot and the quantification of the signals are shown in the figure.

Another key regulator of metabolism and a target of pAMPK is acetyl-CoA carboxylase (ACC), which functions in the production of the malonyl-CoA substrate for the biosynthesis of fatty acids as an alternative energy source [29]. Inhibitory phosphorylation at Ser79, was determined by Western blotting with pACC specific antibodies (Figure 6B). Reducing SELENOF levels resulted in increased phosphorylation of ACC without changes to total ACC levels. Together, the increased phosphorylation of AMPK and ACC when SELENOF levels are reduced indicate a potential function of SELENOF in glycolysis and lipid metabolism.

## 3. Discussion

Loss of SELENOF was considered to be a significant occurrence in prostate cancer progression due to the lower levels in prostate cancers and the association of polymorphisms in the SELENOF gene with prostate cancer mortality [10,15]. In order to extend these observational studies to a model that can begin to assess functional consequences to its loss, SELENOF levels were reduced in the RWPE-1 non-tumorigenic prostate cell line. As a result, these cells acquired the characteristics of the transformed phenotype, including growth in soft agar and increased migration. In addition, the shift to a more aerobic respiration from a glycolytic means of energy production mirrors the changes that happen during the progression of normal prostate epithelium to malignancy. Collectively, these cell culture studies indicate that loss of SELENOF is very likely contributing to disease progression and that loss is not a bystander effect of the carcinogenic process.

Neither the function of SELENOF nor the molecular consequences of its loss are understood. In most cell types, SELENOF is located in the ER, and its location in the ER and tight binding to UGGT, a protein that functions in protein disulfide bond formation and quality control in that organelle, indicates that SELENOF may contribute to that process [9]. In contrast, SELENOF appears to reside in the cell membrane of benign prostatic epithelia based on its co-localization with E-cadherin, being excluded from the apical portion facing the lumen [10]. These observations indicate that SELENOF may have a function in regulating the secretion of bioactive proteins or compounds. Studies with SELENOF knockout mice have indicated the enhanced secretion of non-functional IgM, leading the authors to suggest that SELENOF was a “gatekeeper of secreted disulfide-rich glycoproteins” [30]. Determining whether the loss of SELENOF contributes to prostate cancer progression via a secretory mechanism involving the release of proteins that promote cancer initiation or progression will require additional studies.

The data presented herein along with previous results indicate that the loss of SELENOF during prostate cancer progression may contribute to the disparity in prostate cancer among African American men. The cell culture work provided evidence that lower levels of SELENOF have a physiological impact on prostate epithelial cells. Additionally, prostatic cancers seen in African American men have a lower level of SELENOF [10] with a greater difference between the tumor and benign tissue than Caucasian men. Moreover, the haplotype much more frequent among the genomes of African Americans is predicted to result in less SELENOF based on cell culture studies with reporter constructs that specifically determine the impact of these variations on the recognition of in-frame UGA codons as selenocysteine [8,11]. In addition, SELENOF levels are affected by selenium availability, and this is likely to occur to a greater extent among those with the at-risk haplotype, and several reports have indicated that African Americans exhibit lower levels of selenium [10,31]. The loss of SELENOF in prostate cancers may therefore be a risk factor present disproportionally among the genomes of African Americans that can help guide clinicians in their decisions regarding treatment options. Restoring SELENOF levels may also be developed as a new therapy to treat men with the detrimental genetic variation or greatest loss of SELENOF during cancer progression. Rather than an approach to increasing SELENOF levels using selenium supplementation, we suggest that future studies address the reasons for SELENOF loss in prostate cancers to develop a targeted approach, given concerns raised about selenium supplementation and the potential for enhancing the risk of cancer and other diseases [32,33,34].

## 4. Materials and Methods

Tissue Microarray (TMA). The “120 Case High Grade Race Disparity TMA” was obtained from the Prostate Cancer Biorepository Network (PCBN). This TMA contains tumor and matched benign prostate tissue from 60 self-identified Caucasian and 60 self-identified African American patients matched by age +/− 3 years, Gleason grade, and stage and is enriched for cases with a Gleason score ≥8.

Cell Culture and Plasmid Construction. Immortalized non-transformed RWPE-1 prostate epithelial cells were obtained from ATCC and maintained in keratinocyte serum free media (Gibco. Langley, OK, USA). The media was supplemented with recombinant epidermal growth fact (rEGF) and bovine pituitary extract (BPE). The PC-3 human prostate carcinoma cell line was maintained in RPMI-1640 media (Gibco, Cat#: 11,875) supplemented with 10% fetal bovine serum (Gemini Bio, West Sacramento, CA, USA), 100 U/mL penicillin and 100 μg/mL streptomycin (Gibco). All cells were maintained at 37 °C with 5% CO_2_. Identity verification of the cell lines was performed by Genetica Cell Line Testing (Burlington, NJ, USA) and the Clinical Molecular Pathology Laboratory at UIC by short tandem repeat (STR) analyses. SELENOF shRNA and control scramble RNA were purchased from Origene Technologies (Cat#: TG316,856) and transfected into RWPE-1 cells using ContinuumTM transfection reagent (Gemini Bio, Cat#: 400–700). Transfected cells were selected in 1 μg/mL puromycin (Sigma-Aldrich, St. Louis, MO, USA), individual clones were isolated and expanded for RWPE-1 scramble shRNA transfected cells and RWPE-1 shRNA cells.

Quantification of SELENOF mRNA. Cells were allowed to grow to 90% confluency and total RNA was extracted using the RNeasy Plus Mini Kit (Qiagen, Hilden, Germany, Cat#: 74134) according to the manufacturer’s protocol. The RNA was reverse-transcribed using the high-capacity cDNA reverse transcription kit (Thermofisher, Waltham, MA, USA, Cat#: 4368814) without changes to the protocol. Utilizing the Fast SYBR Green Master Mix (Thermofisher, Cat#: 4,385,612) and QuantStudio 6 Flex Real-time PCR System (Thermofisher), fold changes were calculated using the delta–delta CT method using GAPDH as the internal amplification control. SELENOF was amplified using 6 pmol of forward (5′–TGCGGAAAATGGTAGCGAT–3′) and reverse (5′–CATGCCTCCGATGAAAACTCT–3′) primers per reaction. GAPDH was amplified as an internal control using 6 pmol of forward (5′–ACCCCTTCATTGACCTCAACTA–3′) and reverse (5′–ATCGCCCCACTTGATTTTG–3′) primers.

Western Blotting. Cells were grown to approximately 90% confluency, harvested, and lysed using 1x cell lysis buffer (Cell Signaling Technology, Danvers, MA, USA) containing both protease and phosphatase inhibitors (Millipore). Protein concentrations were measured using the Bradford assay (Bio-Rad, Hercules, CA, USA) and measured using a spectrophotometer (Bio-Rad). Lysates were combined with NuPAGE LDS loading buffer (Life Technologies, Carlsbad, CA, USA) and 1x reducing agent (Life Technologies) and boiled at 95 °C for 10 min. The prepared lysates were electrophoresed on a gradient 4–12% Bis-Tris denaturing polyacrylamide gel (Life Technologies). After electrophoresis, proteins were transferred to a small pore nitrocellulose membrane (Thermofisher). Membranes were blocked using Licor blocking buffer (LI-COR, Lincoln, NE, USA) for 1 h and incubated overnight with antibodies at 4 °C. SELENOF antibody (Abcam, Waltham, MA, USA) was used at 1:2000, GAPDH (Cell Signaling Technologies, Cat#: 2118) at 1:10,000, β-actin (Abcam) at 1:10,000, pAMPKThr172 at 1:1000 (Cell Signaling Technologies, Cat#: 2535), AMPKα at 1:1000 (Cell Signaling Technologies, Cat#: 5832), pACCS79 at 1:1000 (Cell Signaling Technologies, Cat#: 11,818), ACC at 1:1000 (Cell Signaling Technologies, Cat#: 3676) concentrations. Secondary antibodies, either anti-rabbit or anti-mouse (LI-COR Biosciences), were used at 1:5000 concentrations. The membrane was visualized and analyzed using the Odyssey^®^ CLx imaging system (LI-COR Biosciences).

Cell Proliferation and Growth in Semi-Solid Media. Proliferation was measured using the FluoReporter blue fluorometric dsDNA quantitation kit (Thermofisher, Cat#: F-2962). An equal number of cells (5000 cells/well) were plated on a 96-well plate (Corning Inc., Glendale, AZ, USA) in triplicate and were incubated at 37 °C for 3 days according to the manufacturer’s protocol. Growth in semi-sold agar was assayed using a 6-well plate with an equal number of cells in each well (5000 cells/well) containing 0.6% agar (Gibco) and culture media. Cells were incubated at 37 °C for 6 weeks, imaged using an EVOS FL Imaging System (Invitrogen, Waltham, MA, USA and counted using Celeste software (Invitrogen).

Wound Healing Assay. Cells plated for the wound healing or scratch assays were incubated on a 6-well plate until 100% confluency. The wound or scratch was generated by dragging a pipette tip through the middle of the well across the monolayer of cells. The media was replaced after the scratch was created. Aphidicolin (Cayman Chemical, Cat#: 14,007, Ann Arbor, MI, USA), a cell proliferation inhibitor, was added at 1 μ/mL to each of the wells. Images of the wound were captured using an EVOS FL imaging system (Invitrogen, Waltham, MA) at 0 h, 24 h, and 48 h after the initial scratch. Three areas of the wound closure were measured using ImageJ and averaged for each of the conditions.

Oxygen Consumption. Oxygen consumption rate (OCR) was used as a surrogate for oxidative phosphorylation using a Seahorse XF analyzer and Seahorse XF cell mito stress test kit (Agilent Technologies, Inc., Santa Clara, CA, USA) according to the manufacturer’s protocol. In short, oxygen consumption was measured by a fluorophore that is quenched by the presence of oxygen. As mitochondrial respiration consumes oxygen, the signal is measured by the instrument. The cell mito stress test kit utilizes specific electron transport chain inhibitors, oligomycin, carbonyl cyanide 4-(trifluoromethoxy)phenylhydrazone (FCCP), and a combination of rotenone and antimycin A to measure various parameters of mitochondrial function with OCR. Basal measurements are first obtained, followed by sequential injections of these inhibitors. Oligomycin is an ATP synthase or complex V inhibitor that decreases the electron flow through the electron transport chain and builds a gradient for the next injection. This measurement after the oligomycin injection is indicative of mitochondrial ATP production. Next, FCCP, an uncoupler of oxidative phosphorylation is injected and disrupts the proton gradient across the mitochondrial membrane. This enables electrons to flow freely, and this parameter is indicative of the maximal respiration of the mitochondria. The difference between maximal respiration and the initial basal measurement is the spare respiratory capacity or the ability of the mitochondria to respond to various increased energy needs of the cell. Lastly, the combination of rotenone and antimycin A, inhibitors of complex I and III respectively, shuts down mitochondrial respiration completely permitting the measurement of non-mitochondrial respiration in the cells.

Immunohistochemistry. Immunofluorescent staining of the TMA was performed by the UIC Research Histology Core using the BondTM Research Detection System (Leica Biosystems, Buffalo Grove, IL, USA, DS9455). A Leica BondTM (Leica Biosystems, Buffalo Grove, IL, USA) was used for deparaffinization, antigen retrieval, and staining. The tissues were washed with bond dewax solution (Leica Biosystems, AR9222) at 72 °C and then washed with 100% ethanol. Following the ethanol wash and washes with bond wash solution (Leica Biosystems, AR9590), target antigens were unmasked by incubation in bond ER 1 solution at pH 6.0 for 40 min at 98 °C (Leica Biosystems, AR9640). After the incubation with a background sniper protein block (Biocare Medical, #BS966) for 30 min at RT, sequential immunostaining was performed with antibodies directed against SELENOF (Abcam, Cat#: ab124,840, rabbit monoclonal NCIR128A, 1:100 dilution) and E-cadherin (Cell Signaling, Cat#: 14,472, mouse monoclonal 4A2, 1:100). Sections were incubated with the primary antibodies at room temperature for 1 h followed by incubation with Alexa-488 and Alexa-555-conjugated secTondary goat anti-mouse and goat-anti rabbit IgG (Thermofisher Scientific, # A32,734 and #A32,732) at room temperature for 1 h. Lastly, the slides were counterstained with DAPI and mounted with Pro-GoldTM diamond antifade mountant (Thermofisher Scientific, #P36961). Stained tissue slides were scanned at 20x on Vectra 3 automated quantitative imaging system (Akoya Biosciences, Marlborough, MA, USA) and analyzed as previously described [10] by the UIC Research Tissue Imaging Core. Briefly, images were spectrally unmixed and adjusted for tissue autofluorescence. E-cadherin staining was used for tissue segmentation, and after cell segmentation, the levels of SELENOF in epithelial cells were analyzed for each core. Various artifacts were manually excluded.

Statistical Analysis. Statistical analyses were performed in SAS 9.4 by our statistical team. SELENOF levels and percentages in cancer and benign tissues from African American and Caucasian men were compared using paired *t*-tests for within-individual type of tissue comparison or independent group *t*-tests for race comparison. Due to skewness in the observed individual SELENOF levels, log-transformed SELENOF measures were used in all *t*-tests. Multivariate ordinal logistic regression models for Gleason sum categories were employed to assess the association between SELENOF levels, race (African Americans vs. Caucasian), and the potential interactions between them. Two sets of SELENOF percent measures were considered separately in the regression models: 1. SELENOF percentages in cancer and benign cells; the benign minus cancer differences in SELENOF percentages. Interactions between race and SELENOF percent or benign-cancer differences were first tested. Then backward selections were performed for each main SELENOF effects keeping the significant interaction terms in the model. Proportional odds assumptions were tested for each ordinal logistic regression model. In the results, estimate of effects, standard errors, odds ratios with 95% confidence intervals were reported. All statistical tests are two-sided tests that control for a Type I error probability of 0.05. All other experimental data were collected from at least three biologically independent experiments. Results are reported as mean ± SEM and *p* < 0.05 were considered statistically significant.

## 5. Conclusions

In conclusion, the data provided here indicates that SELENOF is a prostate cancer tumor suppressor able to alter the transformed phenotype of human prostate epithelial cells and may contribute to the disparity in prostate cancer mortality that exists among men of African descent.

## Data Availability

All data generated from this study are included in the manuscript and supplemental materials.

## Supplementary Materials

The following are available online at https://www.mdpi.com/article/10.3390/ijms222112040/s1.
