# Supplementary material for: Loss of SELENOF Induces the Transformed Phenotype in Human Immortalized Prostate Epithelial Cells
Source: Int J Mol Sci. 2021 Nov 7;22(21):12040. doi: 10.3390/ijms222112040 (PMC8584825; doi:10.3390/ijms222112040)
Supplement: Supplementary file 1 [file ijms-22-12040-s001.zip › ijms-1422133-supplementary.pdf]

Supplemental Tables

Supplemental Table S1. Mean (SD) of SELENOF Intensities by Tissue Type and Race:

	**Cancer Tissue**			**Benign Tissue**			**Benign - Cancer Difference**		
	Mean (SD)			Mean (SD)			Mean (SD)		p-value
Overall Sample N=119									
Nuc Mean	0.12 (0.22)			0.84 (0.99)			0.72 (0.95)		<0.0001
Cyt Mean	0.1 (0.19)			0.76 (1.03)			0.67 (0.99)		<0.0001
Mem Mean	0.18 (0.3)			1.18 (1.39)			1.01 (1.33)		<0.0001
Cell Mean	0.13 (0.23)			0.87 (1.06)			0.76 (1.02)		<0.0001
Prct Nuc Pos	1.84 (4.61)			15.89 (18.97)			14.17 (18.26)		<0.0001
Prct Cyt Pos	1.41 (3.67)			12.98 (16.67)			11.67 (16.2)		<0.0001
Prct Mem Pos	2.67 (6.81)			22 (22.59)			19.49 (22.05)		<0.0001
Prct Cells Pos	1.81 (4.71)			16.19 (19.68)			14.5 (19.08)		<0.0001
By Race African American N=60; Caucasian N=59;									
	African American	Caucasian	p-value	African American	Caucasian	p-value	African American	Caucasian	p-value
Nuc Mean	0.16 (0.26)	0.08 (0.15)	0.045	0.96 (1.05)	0.73 (0.93)	0.18	0.8 (0.99)	0.65 (0.91)	0.40
Cyt Mean	0.14 (0.24)	0.06 (0.12)	0.036	0.86 (1.09)	0.67 (0.97)	0.24	0.72 (1.03)	0.61 (0.96)	0.52
Mem Mean	0.24 (0.38)	0.11 (0.18)	0.024	1.35 (1.49)	1.02 (1.27)	0.17	1.12 (1.42)	0.91 (1.24)	0.40
Cell Mean	0.17 (0.28)	0.08 (0.15)	0.036	1 (1.12)	0.76 (0.99)	0.19	0.83 (1.06)	0.68 (0.97)	0.43
Prct Nuc Pos	2.59 (5.55)	1.08 (3.29)	0.038	18.26 (20.0)	13.72 (17.75)	0.22	15.67 (19.48)	12.62 (16.94)	0.37
Prct Cyt Pos	2.07 (4.67)	0.73 (2.07)	0.047	14.97 (17.7)	11.14 (15.51)	0.22	12.9 (17.06)	10.39 (15.3)	0.40
Prct Mem Pos	3.87 (8.64)	1.45 (3.93)	0.039	25.2 (23.86)	19.06 (20.95)	0.23	21.34 (23.72)	17.58 (20.21)	0.36
Prct Cells Pos	2.62 (5.83)	0.99 (3.02)	0.036	18.62 (20.9)	13.95 (18.26)	0.23	16 (20.41)	12.94 (17.64)	0.39

Note: All p-values were from Log-transformed data due to skewness in the observed distribution.

Supplemental Table S2a. SELENOF Percent in Benign and Cancer Cells as Covariates:

	**Estimate (SE)**	**p-value**	**Odds Ratio (95% CI)**
**Benign Cyt Prct**	-0.26 (0.07)	0.0003	0.92 (0.86, 0.98)
**Race: Cauc as reference African Americans**	-0.12 (0.39)	0.75	OR for Cancer Mem Prct in Caucasians: 0.71 (0.59, 0.87) OR for Cancer Mem Prct in African Americans: 0.92 (0.86, 0.98)
**Cancer Mem Prct**	-0.34 (0.10)	0.0006	OR for Cancer Mem Prct in Caucasians: 0.71 (0.59, 0.87) OR for Cancer Mem Prct in African Americans 0.92 (0.86, 0.98)
**Cancer Mem Prct * African Americans**	0.26 (0.10)	0.0132	

S2b. Benign – Cancer SELENOF Percent differences as Covariates:

	**Estimate (SE)**	**p-value**	**Odds Ratio (95% CI)**
**Race: Cauc as reference African Americans**	-0.48 (0.44)	0.28	1.61 (0.26, 1.47)
**Benign – Cancer diff in Cell Prct**	-0.02 (0.08)	0.80	OR for Diff in Cell Prct in Caucasians: 0.98 (0.85, 1.14) OR for Diff in Cell Prct in African Americans 1.57 (1.20, 2.04)
**Benign – Cancer diff in Cell Prct * African Americans**	0.47 (0.15)	0.0024	
**Benign – Cancer diff in Cyt Prct**	-0.001 (0.09)	0.99	OR for Diff in Cyt Prct in Caucasians: 0.999 (0.84, 1.19) OR for Diff in Cyt Prct in African Americans 0.59 (0.43, 0.80)
**Benign – Cancer diff in Cyt Prct * African Americans**	-0.52 (0.18)	0.0033	
